# Sleep‐Related Attentional Bias in Insomnia: A Drift Diffusion Model Approach

**DOI:** 10.1111/jsr.70315

**Published:** 2026-02-24

**Authors:** Isla Tsz Kwan Hui, Tommy Ho‐Fung Chung, Sing‐Hang Cheung, Nazanin Biabani, Panagis Drakatos, Romola S. Bucks, Sharon L. Naismith, David O'Regan, Veena Kumari, Dieter Riemann, Toby Wise, Shirley Xin Li, Ivana Rosenzweig

**Affiliations:** ^1^ Department of Psychology The University of Hong Kong Hong Kong Special Administrative Region Hong Kong; ^2^ Sleep and Brain Plasticity Centre, Department of Neuroimaging Institute of Psychiatry, Psychology and Neuroscience (IoPPN), King's College London London UK; ^3^ Sleep Disorders Centre, Guy's and St Thomas' NHS Foundation Trust London UK; ^4^ School of Basic and Medical Biosciences, Faculty of Life Science and Medicine King's College London London UK; ^5^ School of Psychological Science The University of Western Australia Crawley Australia; ^6^ Healthy Brain Ageing Program, the Brain and Mind Centre University of Sydney Australia; ^7^ Centre for Cognitive and Clinical Neuroscience (CCN) College of Health, Medicine and Life Sciences, Brunel University of London London UK; ^8^ Department of Psychiatry and Psychotherapy, Medical Faculty Freiburg University Medical Center, University of Freiburg Germany; ^9^ Department of Neuroimaging, Institute of Psychiatry Psychology and Neuroscience (IoPPN), King's College London London UK

**Keywords:** anxiety, attentional bias, dot‐probe, drift diffusion model, hyperarousal, insomnia

## Abstract

Cognitive models propose that insomnia is maintained in part by selective attention to sleep‐related information, yet reaction‐time indices alone offer limited mechanistic specificity. We investigated sleep‐related attentional bias in adolescents and young adults with insomnia disorder (*n* = 201; aged 15–24 years; DSM‐5) using a sleep‐related dot‐probe task with sleep‐related and neutral Cantonese Chinese word pairs. Trial‐level responses were analysed with Hierarchical Drift Diffusion Modelling (HDDM) to estimate drift rate (*v*), the speed of evidence accumulation for probe response choices and to examine moderation by anxiety symptoms. Drift rates were higher on congruent than incongruent trials (*q* = 0.036), indicating faster evidence accumulation when the probe appeared in the location of sleep‐related words, consistent with sleep‐related attentional bias indexed indirectly via probe responses. Higher anxiety was associated with faster drift rates across both trial types (*q* = 0.023 and *q* = 0.024), consistent with generalised hyperarousal rather than selective enhancement of sleep‐related bias. The congruency × anxiety interaction was not significant (95% HDI [−0.10, 0.31]). These findings provide computational evidence consistent with sleep‐related attentional bias in young people with insomnia and suggest that comorbid anxiety is associated with broadly increased evidence accumulation rather than cue‐specific amplification.

## 1 Introduction

Insomnia disorder is a highly prevalent and chronic disorder, characterised by persistent difficulties initiating sleep, maintaining sleep and/or early morning awakenings, accompanied by clinically significant distress or daytime impairment (American Academy of Sleep 2014; American Psychiatric Association 2013; Riemann et al. 2022). Epidemiological studies estimate that approximately 10% of the population meets clinical diagnostic criteria, with around 40% of cases persisting beyond 5 years (Morin and Jarrin 2022). Beyond its impact on sleep continuity, insomnia is associated with cognitive impairments, emotional dysregulation and an increased risk of psychiatric disorders, including anxiety and depression(Breslau et al. 1996; Brownlow et al. 2020; Fortier‐Brochu et al. 2010, 2012; Fortier‐Brochu and Morin 2014; Morin and Jarrin 2022; Neckelmann et al. 2007; Riedel and Lichstein 2000; Zhang et al. 2012; O'Regan et al. 2025). Growing evidence suggests that insomnia is maintained by a complex interplay between cognitive biases, cortical hyperarousal and dysregulated neurobiological processes, leading to maladaptive attentional and emotional regulation patterns that perpetuate the disorder (Morin et al. 2015; Riemann et al. 2022; Riemann et al. 2010).

### 1.1 Cognitive Models of Insomnia and Sleep‐Related Attentional Bias

Cognitive models propose that insomnia is perpetuated by distorted attentional processes that bias perception towards sleep‐related stimuli, reinforcing maladaptive cognitive appraisals and pre‐sleep hyperarousal (Espie et al. 2006; Harvey 2002; Perlis et al. 1997; Riemann et al. 2010). Heightened vigilance towards sleep‐related information may lead to excessive monitoring of sleep cues, contributing to the anticipation of poor sleep and increasing nocturnal cognitive and physiological arousal. Attentional bias towards sleep‐related information has been demonstrated in multiple behavioural studies (Akram et al. 2023; Harris et al. 2015), including the use of dot‐probe tasks, where insomnia patients respond more rapidly to sleep‐related stimuli than neutral stimuli, indicating a preferential allocation of attention (MacMahon et al. 2006). However, conventional reaction time analyses lack the sensitivity to differentiate the cognitive mechanisms underlying these biases, leaving uncertainty as to whether they arise from faster evidence accumulation, response bias or differences in decision‐making thresholds.

### 1.2 Theoretical Neuroscientific Models of Insomnia: Hyperarousal and Neurocognitive Dysregulation

Beyond cognitive frameworks, neuroscientific models of insomnia advance alterations in cortical and subcortical circuits involved in arousal regulation, executive control and emotional processing (Perlis et al. 1997; Perlis et al. 2005; Van Someren 2021). The hyperarousal model posits that chronic insomnia is characterised by increased physiological, cognitive and emotional arousal, disrupting the transition to sleep and leading to fragmented sleep architecture (Dressle and Riemann 2023; Perlis et al. 1997; Riemann et al. 2010). Functional neuroimaging studies have demonstrated increased resting‐state activity in the default mode network, as well as heightened activation of the amygdala and anterior cingulate cortex (ACC) in response to emotionally salient stimuli in individuals with insomnia (Baglioni et al. 2014; Dai et al. 2020). The persistence of elevated amygdala reactivity, coupled with impaired prefrontal regulatory control, suggests that individuals with insomnia exhibit a maladaptive attentional bias towards threat‐related and self‐referential information, sustaining hypervigilance and reinforcing dysfunctional cognitive patterns (Huang et al. 2012).

Disruptions in neuroplasticity mechanisms have also been implicated in insomnia, with accumulating evidence linking chronic sleep disturbances to alterations in brain‐derived neurotrophic factor (BDNF) expression (Ballesio et al. 2023). BDNF is a critical modulator of synaptic plasticity and cortical excitability, and its downregulation in insomnia may contribute to cognitive inflexibility and attentional biases (Ballesio et al. 2023; Giese et al. 2014). Lower BDNF levels have been associated with reduced prefrontal inhibition of the limbic system, leading to impaired top‐down regulation of emotional and attentional processes (Wang et al. 2022). This dysregulation may underlie the excessive focus on sleep‐related concerns and the difficulty in disengaging from negative cognitions, reinforcing insomnia‐related hyperarousal.

### 1.3 Computational Modelling and the Drift Diffusion Model (DDM)

**FIGURE 1:**
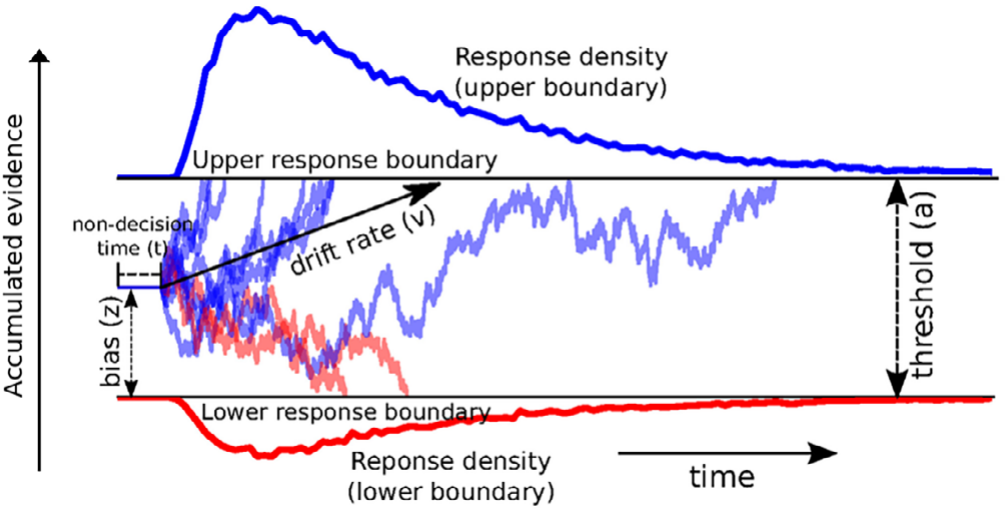
Schematic representation of the drift diffusion model (DDM). The DDM conceptualises two‐choice decision‐making as a noisy process of evidence accumulation over time (*y*‐axis), in which a response is made once the accumulated evidence reaches one of two decision boundaries. Key parameters include: (i) drift rate (*v*), reflecting the speed and direction of evidence accumulation; (ii) boundary separation (*a*), representing the amount of evidence required before making a choice; (iii) starting point (*z*), indexing response bias and (iv) non‐decision time (*t*), reflecting sensory encoding and motor execution time. In dot‐probe paradigms, these parameters index the decision process for the probe response; therefore, differences in drift rate between congruent and incongruent trials provide an indirect index of attentional allocation during the preceding stimulus exposure, consistent with established reaction‐time interpretations of attentional bias. *Source:* Adapted with permission from Wiecki et al. (2013).

Traditional behavioural analyses have been limited in their ability to disentangle the distinct cognitive components underlying attentional bias. The application of computational modelling techniques, such as the Drift Diffusion Model (DDM; see Figure 1), offers a more sophisticated approach to quantify decision‐making processes in insomnia (Wiecki et al. 2013). The HDDM framework decomposes cognitive processes into distinct components, including drift rate, decision threshold and non‐decision time, allowing for a more precise characterisation of attentional bias mechanisms (Ratcliff and McKoon 2008; Ratcliff and Smith 2004). Drift rate, a key parameter of the model, represents the speed at which evidence is accumulated before a decision is made and is particularly relevant for understanding attentional processing in insomnia (Wiecki et al. 2013). By applying DDM to a sleep‐related dot‐probe task, it becomes possible to determine whether insomnia patients exhibit a true cognitive bias towards sleep‐related stimuli, characterised by faster evidence accumulation or whether their attentional processing is influenced by other cognitive biases, such as response conservatism or altered decision thresholds.

### 1.4 HDDM Application to Sleep‐Related Attentional Bias in Insomnia

**FIGURE 2:**
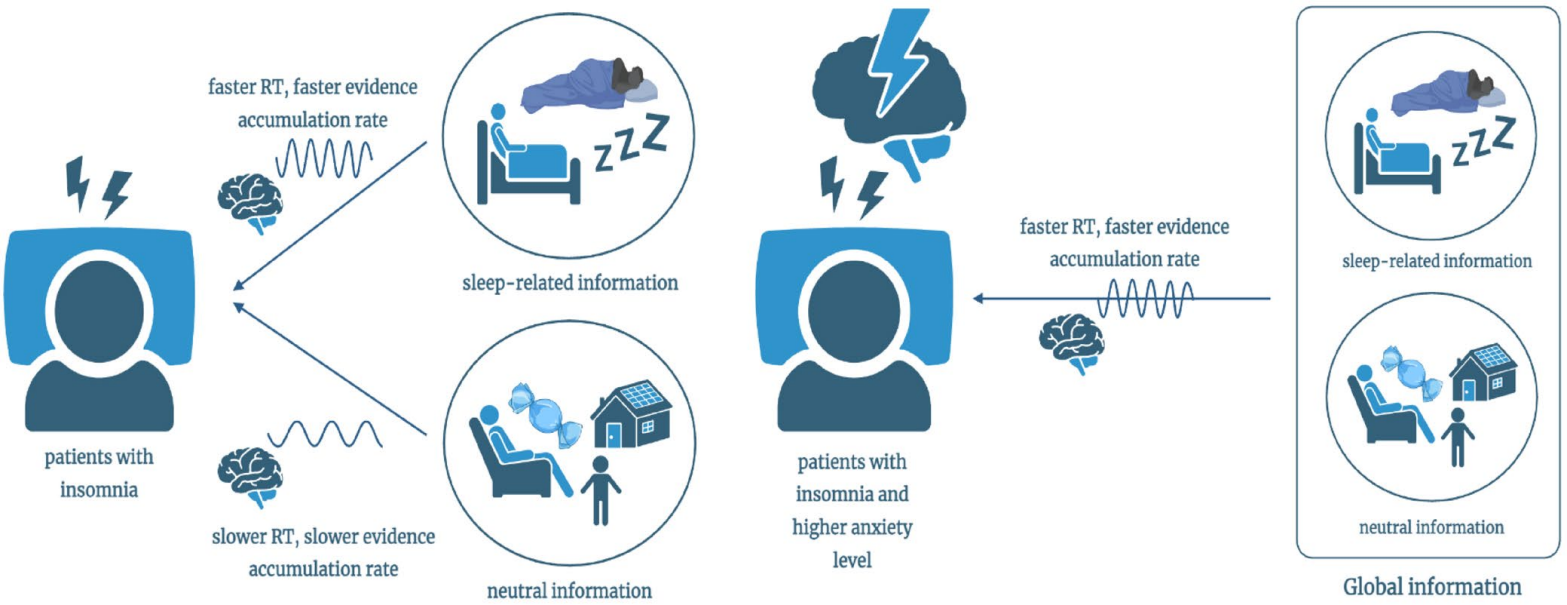
Study overview and principal findings. Participants with insomnia completed a sleep‐related dot‐probe task using sleep‐related and neutral word pairs, and their response times were analysed using the Hierarchical Drift Diffusion Model (HDDM). The model estimated drift rate (*v*), representing the speed of evidence accumulation for probe responses on congruent trials (probe replacing sleep‐related words) versus incongruent trials (probe replacing neutral words). Results showed significantly faster drift rates on congruent trials, consistent with sleep‐related attentional bias indexed indirectly via probe‐related evidence accumulation conditional on stimulus congruency. Individuals with higher anxiety demonstrated faster drift rates across both trial types, consistent with a generalised hyperarousal profile. There was no significant interaction between anxiety and stimulus congruency, suggesting that anxiety did not selectively amplify sleep‐related attentional bias. *Source:* Created in BioRender. Hui (2025) https://BioRender.com/wkv0vsa.

The present study applied HDDM to examine the cognitive mechanisms underlying sleep‐related attentional bias in insomnia (Figure 2). Two primary hypotheses were tested: first, that insomnia patients would exhibit a faster drift rate for probe responses on congruent trials (probe replacing sleep‐related words) than on incongruent trials, indicative of an increased attentional bias towards sleep‐related information; and second, that anxiety symptoms would modulate drift rate, leading to an overall increase in evidence accumulation speed, potentially exacerbating sleep‐related attentional bias.

## 2 Methods and Materials

### 2.1 Participants

A total of 270 individuals with insomnia disorder (Table S1) were recruited for two randomised controlled trials investigating the efficacy of group‐based cognitive behavioural therapy (CBT) for insomnia, with one trial including comorbid depression (Li et al. 2021) and the other targeting individuals with an evening chronotype (Li et al. 2024). Recruitment was conducted via secondary schools, universities and non‐governmental organisations in Hong Kong. The present study utilised only baseline, pre‐treatment data to investigate sleep‐related attentional bias and its association with comorbid anxiety using computational modelling.

All participants met diagnostic criteria for insomnia disorder according to the Diagnostic and Statistical Manual of Mental Disorders, Fifth Edition (DSM‐5) (American Psychiatric Association 2013), and scored > 9 on the Insomnia Severity Index (ISI), a validated threshold for clinically significant insomnia (Chung et al. 2011). Diagnostic confirmation and comorbidity screening were performed using the Diagnostic Interview for Sleep Patterns and Disorders (DISP) (Merikangas et al. 2014) and the Mini International Neuropsychiatric Interview (MINI) (Liu et al. 2011).

Participants ranged in age from 15 to 24 years (*M* = 20.05, SEM = 0.13); 57.2% identified as female. The majority were tertiary‐level students (95.5%). Exclusion criteria included night shift work, current use of prescribed hypnotics or other medications taken specifically to facilitate sleep (including melatonin), and a history of psychotic, neurodevelopmental or sleep disorders (other than insomnia). Stable psychotropic medications prescribed for psychiatric indications (e.g., antidepressants) and other long‐term medications were permitted and recorded at baseline. Comorbid psychiatric conditions were prevalent: 37.8% met criteria for major depressive disorder, 20.9% for generalised anxiety disorder and smaller proportions for dysthymia, social anxiety disorder and obsessive‐compulsive disorder. Written informed consent was obtained from all participants (and from parents of minors), and the study was approved by the Human Research Ethics Committee of the University of Hong Kong.

### 2.2 Assessment of Insomnia and Anxiety Symptoms

Insomnia symptom severity was measured using the ISI, a 7‐item instrument rated on a 5‐point Likert scale (0–4), assessing the nature, severity and impact of insomnia symptoms over the past 2 weeks. Anxiety symptoms were assessed using the 7‐item Anxiety subscale of the Hospital Anxiety and Depression Scale (HADS‐A) (Zigmond and Snaith 1983), which evaluates self‐reported anxiety over the preceding week. Higher scores on both instruments reflect greater symptom severity. The ISI and HADS‐A have been previously validated in the Hong Kong population and demonstrated good internal consistency in the current sample (Cronbach's *α* = 0.741 and 0.863, respectively) (Chan et al. 2010; Chung et al. 2011).

### 2.3 Sleep‐Related Dot‐Probe Task

Sleep‐related attentional bias was assessed using a Cantonese Chinese adaptation of the dot‐probe task, originally developed by MacLeod et al. (1986), and subsequently modified to examine sleep‐relevant processing biases. The task involved 20 sleep‐related and 20 neutral word stimuli, drawn from established stimulus sets (MacMahon et al. 2006), and matched on number of Chinese character strokes and frequency of usage in spontaneous Cantonese speech (see Tables S2 and S3). Stimuli were translated and adapted using lexical data from the Hong Kong Cantonese Corpus (Luke and Wong 2015). Independent *t*‐tests confirmed no significant differences between word types in stroke count (*p* = 0.586) or frequency of occurrence (*p* = 0.234).

Each trial began with a 300 ms fixation cross, followed by a 1000 ms presentation of a word pair (one sleep‐related, one neutral) in upper and lower screen positions. Immediately thereafter, a probe (a white dot) appeared in the location of one of the words. Participants were instructed to indicate the location of the dot by pressing the corresponding arrow key. Trials were categorised as congruent if the probe replaced a sleep‐related word and incongruent if it replaced a neutral word. Stimulus positions were counterbalanced and trial order randomised to control for order effects.

**FIGURE 3:**
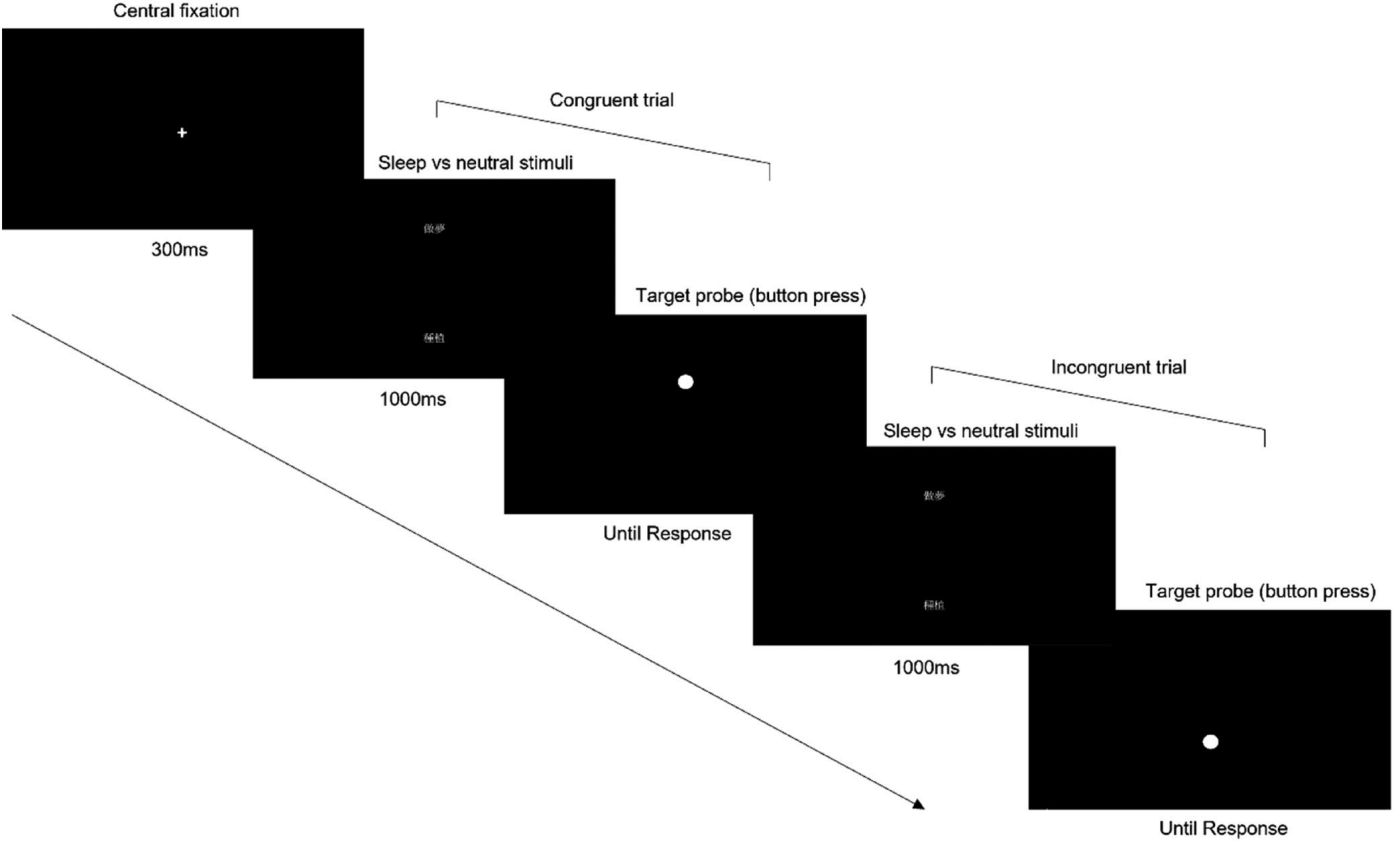
Illustration of the sleep‐related dot‐probe task. Each trial began with a 300 ms central fixation cross, followed by the presentation of a word pair (one sleep‐related and one neutral word) for 1000 ms. A probe (white dot) then appeared in the location of one of the previously presented words. Participants responded by indicating the dot's location via keyboard input. Trials were classified as congruent when the probe replaced a sleep‐related word and incongruent when it replaced a neutral word. The task comprised 40 experimental and 8 practise trials, with all word pairs counterbalanced across position and trial type. The task was implemented in E‐Prime (*Version 2.0*).

The task comprised 40 experimental trials and 8 practise trials. Each word pair was presented twice. No response time limit was imposed. The task was administered using E‐Prime software (*Version 2.0, Psychology Software Tools*) (Schneider et al. 2002); see Figure 3.

### 2.4 Analytical Approach

#### 2.4.1 Behavioural Data Processing and Group Comparisons

Demographic and clinical variables were summarised using means (±SEM) or counts (%). Between‐group comparisons (low vs. high anxiety) were conducted using Mann–Whitney U tests for continuous variables and Pearson's χ^2^ tests for categorical variables, due to non‐normal distributions. For reaction time (RT) data from the dot‐probe task, error trials and responses exceeding ±3 standard deviations from each participant's mean RT were excluded. Participants with > 5% error rates were also excluded. Behavioural congruency analyses were conducted on participants with complete RT data in both conditions. An attentional bias interference score (AB score) was calculated as: (RT_Incongruent − RT_Congruent)/2, with positive values reflecting attentional vigilance towards sleep‐related stimuli and negative values indicating avoidance (MacMahon et al. 2006). A paired‐samples *t*‐test was conducted to assess within‐subject RT differences between congruent and incongruent conditions. Statistical significance was set at *p* < 0.05. All behavioural analyses were conducted using R version 4.4.1 (R Core Team 2024).

#### 2.4.2 Computational Modelling: Hierarchical Drift Diffusion Model (HDDM)

Hierarchical Bayesian estimation of the drift diffusion model was implemented using the DockerHDDM package (Pan et al. 2022; Wiecki et al. 2013) within a Docker environment (Merkel 2014). HDDM fits the model to trial‐level reaction‐time distributions and probe response choices, jointly estimating core parameters including drift rate (*v*), boundary separation (*a*), starting point (*z*) and non‐decision time (*t*). In the dot‐probe paradigm, these parameters index the decision process for the probe response; consequently, differences in *v* between congruent and incongruent trials provide an indirect index of attentional allocation during the preceding stimulus exposure, consistent with established reaction‐time interpretations of attentional bias. Posterior distributions were generated using Markov Chain Monte Carlo (MCMC) sampling with 50,000 draws across four chains, discarding the first 15,000 as burn‐in. Convergence was assessed via visual inspection of trace and autocorrelation plots, and *r*‐hat statistics (threshold < 1.1) (Gelman and Rubin 1992).

A pre‐specified three‐step model‐fitting strategy was employed. First, a baseline model (Model 1) estimated drift rate (*v*) for all participants across congruent and incongruent trials. Next, participants were stratified by anxiety severity (HADS‐A cut‐off ≥ 8) into low anxiety (*n* = 64) and high anxiety (*n* = 137) subgroups. Separate models (Model 2 and Model 3) were fit to compare drift rates within each subgroup. Finally, to assess an interaction between anxiety and trial type (congruency), a multilevel regression model (Model 4) was fit using the formula: *v* = 1 + Congruency + Anxiety + Congruency × Anxiety.

Posterior distributions were interpreted using Bayesian thresholds: a *q*‐value < 0.05 was considered meaningful for *Models 1–3*, while in *Model 4*, a 95% Highest Density Interval (HDI) not containing zero was considered evidence of an effect. Although some prior studies have explored non‐decision time (*t*) in attentional bias tasks (Price et al. 2019), this parameter reflects peripheral processes (e.g., motor execution) (Ratcliff and McKoon 2008; Wiecki et al. 2013) and was not central to the present hypotheses. Therefore, only drift rate (*v*) was modelled as a function of congruency and anxiety, while boundary separation (*a*), starting bias (*z*), and non‐decision time (*t*) were estimated at the individual level. All models used stimulus coding to reflect ongoing evidence accumulation towards discrete response options.

## 3 Results

### 3.1 Sample Characteristics

Of the 270 participants initially recruited, 201 individuals (aged 15–24 years) were included in the final analyses. Exclusions were due to incomplete dot‐probe task data (*n* = 19) or missing questionnaire responses (*n* = 50). No participants met the > 5% error‐rate exclusion criterion. Medication use was not associated with RT performance (linear regression, *p* = 0.455). For full demographic and other clinical characteristics, see Table S1. For behavioural RT analyses of congruency effects, one participant had missing RT data for the congruent condition; therefore, paired RT and AB‐score analyses were based on *n* = 200 participants with complete data for both conditions.

### 3.2 Behavioural Evidence of Sleep‐Related Attentional Bias

Participants responded slightly faster on congruent trials (*M* = 0.407 s, SD = 0.060) than on incongruent trials (*M* = 0.410 s, SD = 0.059), indicating a small behavioural trend in the expected direction. Here, SDs reflect between‐participant variability in participants' mean RTs for each condition. The congruency effect was small and did not reach conventional statistical significance (paired *t*‐test: *t* = 1.949, df = 199, *p* = 0.053; d_av = 0.045; *n* = 200).

### 3.3 Drift Diffusion Modelling Results

**FIGURE 4:**
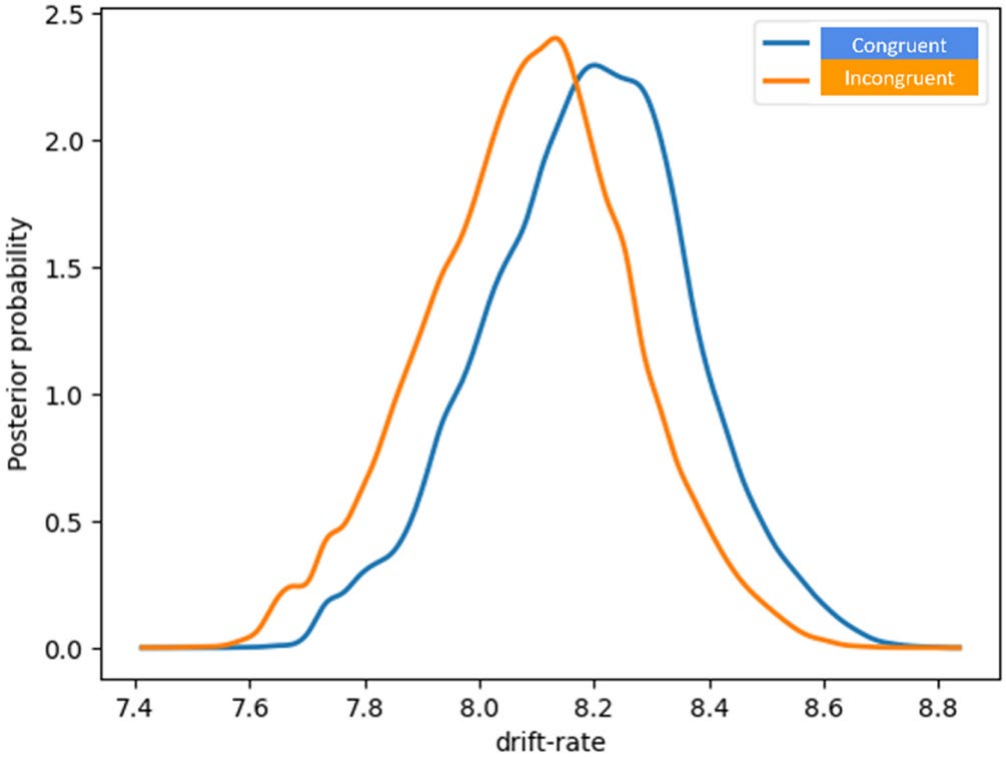
Posterior distributions of drift rate (*v*) across congruent and incongruent trials for all participants (Model 1). Hierarchical Drift Diffusion Modelling (HDDM) revealed significantly faster drift rates on congruent trials (probe replacing sleep‐related words) compared with incongruent trials (probe replacing neutral words) (*q* = 0.036). This pattern is consistent with sleep‐related attentional bias indexed indirectly via probe‐related evidence accumulation conditional on stimulus congruency. Curves show kernel density estimates of the posterior distributions.

**FIGURE 5:**
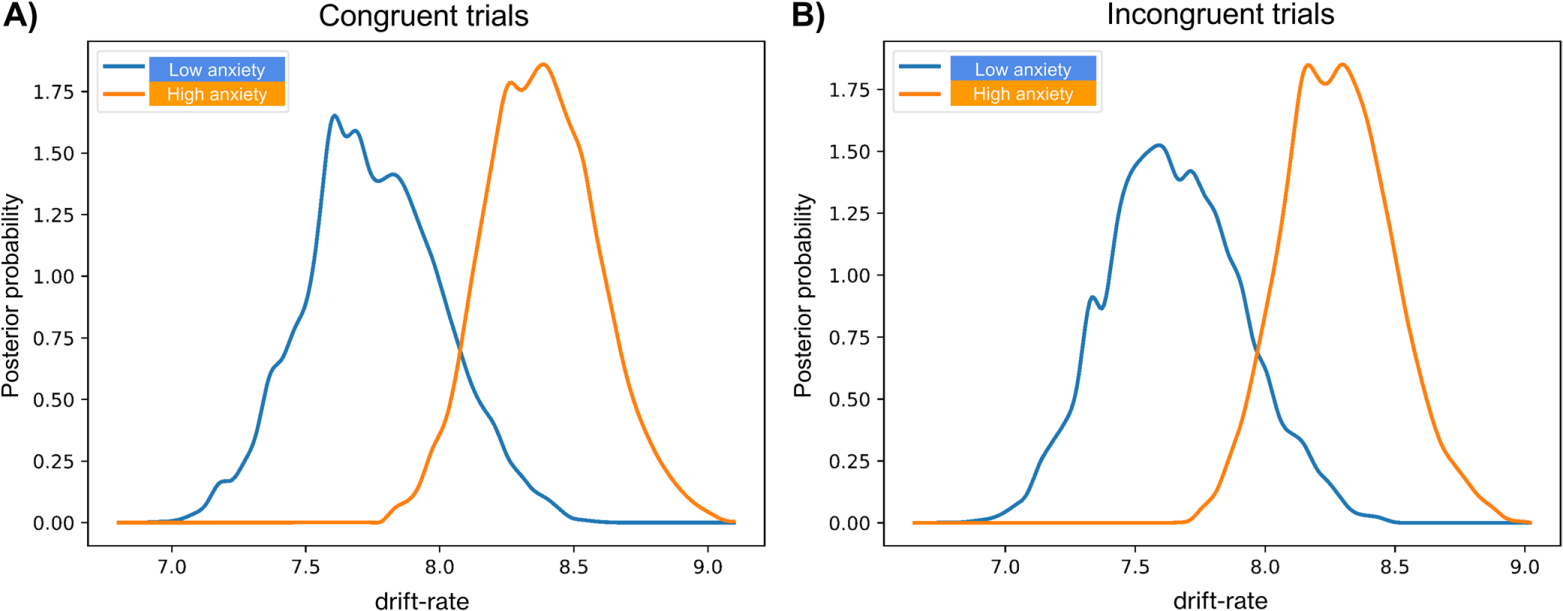
Posterior drift rate distributions stratified by anxiety severity (*Models 2 and 3*). (A) Participants with high anxiety (HADS‐A ≥ 8) exhibited significantly faster drift rates for congruent trials compared to low‐anxiety participants (*q* = 0.023). (B) A similar effect was observed for incongruent trials (*q* = 0.024), suggesting a generalised increase in attentional engagement rather than a cue‐specific effect. These findings support a hyperarousal profile associated with elevated anxiety in insomnia. Posterior distributions reflect group‐level parameter estimates.

**FIGURE 6:**
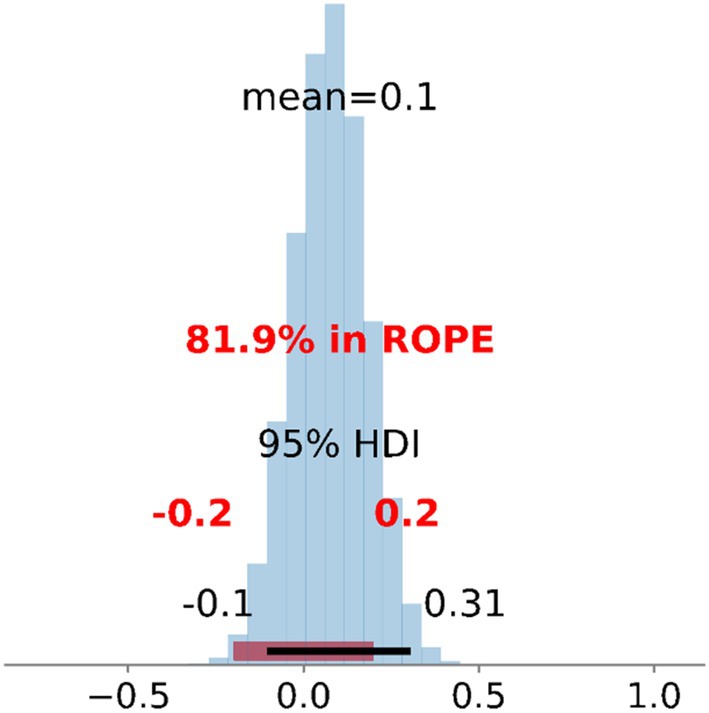
Interaction effect of congruency and anxiety on drift rate (*Model 4*). Posterior distribution of the interaction term from the regression model (*v* ~ Congruency × Anxiety) indicates no significant effect, with the 95% HDI encompassing zero (HDI = [−0.10, 0.31]). This suggests that anxiety severity did not significantly modulate the difference in drift rate between congruent and incongruent trials, consistent with a non‐specific attentional enhancement in high‐anxiety individuals.

All hierarchical Bayesian models achieved satisfactory convergence (Figures 4, 5, 6).

#### 3.3.1 Model 1: Main Effect of Congruency

Across the full sample, drift rates were significantly higher on congruent trials (probe replacing sleep‐related words) compared with incongruent trials (probe replacing neutral words) (*q* = 0.036), indicating more efficient probe‐related evidence accumulation when the probe replaced sleep‐related words (see Figure 4). This supports the hypothesis of a sleep‐specific attentional bias at the level of evidence accumulation.

#### 3.3.2 Models 2 and 3: Drift Rate by Anxiety Subgroup

In subgroup analyses, participants with high anxiety (HADS‐A ≥ 8) exhibited significantly elevated drift rates across both congruent (*q* = 0.023; see Figure 5A) and incongruent trials (*q* = 0.024; see Figure 5B) relative to those with lower anxiety. This pattern indicates a generalised increase in evidence accumulation speed, consistent with a hyperarousal profile.

#### 3.3.3 Model 4: Congruency and Anxiety Interaction

The interaction between congruency and anxiety level was not significant (95% Highest Density Interval [HDI] = [−0.10, 0.31]; see Figure 6), indicating that elevated anxiety did not further amplify sleep‐specific attentional bias. Rather, anxiety was associated with non‐specific enhancements in attentional processing across conditions.

## 4 Discussion

This study provides novel mechanistic insights into sleep‐related attentional processing in insomnia, using a computational framework that expands the resolution of conventional behavioural analysis. By applying Hierarchical Drift Diffusion Modelling (HDDM) to a dot‐probe paradigm, we were able to isolate drift rate, an index of evidence accumulation speed, as a cognitive marker of attentional engagement. Importantly, in the dot‐probe task, this effect reflects probe‐related evidence accumulation conditional on stimulus congruency, providing an indirect but mechanistically interpretable marker of attentional allocation. Our findings confirm that individuals with insomnia exhibit faster drift rates on congruent trials (probe replacing sleep‐related words), consistent with a disorder‐specific attentional bias. In addition, we demonstrate that elevated anxiety symptoms are associated with globally accelerated drift rates across both congruent and incongruent trials, indicating a generalised hypervigilant attentional profile rather than a selective amplification of sleep‐related processing.

### 4.1 Sleep‐Related Attentional Bias and Cognitive Dysregulation

These results extend previous findings in the insomnia literature by offering a more precise decomposition of decision‐making dynamics during attentional tasks (Whitney et al. 2019). Whereas RT‐based approaches aggregate multiple cognitive processes into a single measure, HDDM permits the dissociation of evidence accumulation from motor execution and decision threshold (Wiecki et al. 2013). Our findings suggest that sleep‐related attentional bias in insomnia is indexed by enhanced evidence accumulation, as reflected in drift rate.

This selective increase in drift rate may reflect heightened cognitive salience assigned to sleep‐related cues, potentially reinforcing maladaptive schemas and anticipatory worry that perpetuate insomnia (Riemann et al. 2022). These results are consistent with cognitive‐behavioural models, which posit that insomnia is maintained by selective attention to internal and external signals of arousal or sleep threat, fuelling a cycle of hypervigilance and sleep effort (Espie et al. 2006; Harvey 2002; Riemann et al. 2010). The observed attentional bias may serve as a cognitive correlate of the ‘attention–intention–effort’ pathway proposed by Espie et al. (2006), wherein excessive monitoring of sleep cues leads to escalating pre‐sleep arousal.

From a neurobiological perspective, these attentional patterns likely emerge from impaired prefrontal regulatory control over subcortical salience networks, particularly the amygdala and the bed nucleus of the stria terminalis (Kenwood et al. 2022; Lebow and Chen 2016; Šimić et al. 2021). This circuitry has been implicated in both threat interpretation and sleep–wake regulation (Giardino and Pomrenze 2021; Lebow and Chen 2016). The shift in attentional dynamics towards sleep‐related cues may reflect a failure of cortical inhibition over bottom‐up limbic activation, resulting in enhanced threat sensitivity and cognitive arousal even in the absence of immediate stressors (Calhoon and Tye 2015).

Additionally, neuroplasticity‐related factors may underlie these cognitive signatures. BDNF plays a critical role in modulating synaptic efficacy and cortical excitability, and its downregulation has been consistently associated with chronic insomnia (Ballesio et al. 2023). Recent work highlights how BDNF–TrkB (tropomyosin receptor kinase B) signalling influences local slow‐wave expression and sleep pressure, particularly via Layer 5 pyramidal neurons (ElGrawani et al. 2024). Reductions in BDNF may impair the flexibility of attentional networks, leading to persistent, schema‐driven biases towards disorder‐relevant stimuli. Although BDNF was not measured in the present study, these mechanistic insights may offer a plausible neurobiological substrate for the attentional inflexibility we observed.

### 4.2 Anxiety, Hyperarousal and Generalised Evidence Accumulation

Our second principal finding was that anxiety symptoms were associated with a generalised increase in drift rate across all trial types, irrespective of stimulus congruency. This suggests that anxiety contributes to a non‐specific increase in attentional reactivity, rather than an amplified sensitivity to sleep‐related cues per se. This pattern is consistent with hyperarousal models of insomnia and aligns with transdiagnostic conceptualisations of anxiety as a disorder of over‐interpretation and evaluation of ambiguous stimuli (Riemann et al. 2010).

Circuit‐level models of anxiety point to distributed dysfunction across corticolimbic networks, particularly the amygdala, medial prefrontal cortex and ventral hippocampus, which support the integration and evaluation of environmental threats (Calhoon and Tye 2015). It has been demonstrated that anxiety arises from imbalance within these circuits, where interpretive bias in the amygdala and BNST is insufficiently regulated by top‐down prefrontal activity (Calhoon and Tye 2015). Our observed pattern of global drift rate elevation in high‐anxiety individuals is concordant with this view, suggesting a network‐level increase in interpretive salience rather than a cue‐specific attentional shift.

Of note, this diffuse attentional engagement in high‐anxiety insomnia patients may reflect an altered set‐point of the brain's threat‐evaluation system. In individuals with chronic anxiety, limbic pathways are more likely to assign emotional value to neutral cues, leading to a persistent vigilance state (Calhoon and Tye 2015). This is consistent with findings from functional magnetic resonance imaging and electrophysiological studies indicating tonic hyperactivation of the amygdala and deficient engagement of medial prefrontal regions during emotional regulation tasks in anxiety populations (Calhoon and Tye 2015).

### 4.3 Absence of Anxiety × Congruency Interaction: Developmental and Circuit‐Level Interpretations

While we hypothesised that anxiety would exacerbate sleep‐related attentional bias, our results did not support a significant interaction between anxiety severity and stimulus congruency. Several possible interpretations merit consideration. First, a ceiling effect may be present, whereby the magnitude of sleep‐related attentional bias in insomnia is already maximised and not amenable to further amplification. Prior work has demonstrated small‐to‐moderate effect sizes for such biases (Harris et al. 2015), suggesting their partial saturation in clinical samples.

Second, it is possible that the anxiety experienced by participants was not sufficiently cue‐specific. Whereas some models emphasise sleep‐related worry as a core feature of insomnia, emerging evidence indicates that many patients exhibit broader patterns of ruminative and generalised worry, particularly those with comorbid anxiety disorders (Baglioni et al. 2014; Grupe and Nitschke 2013; Palagini et al. 2024). Such generalised cognitive hyperactivity may blunt the relative distinction between sleep‐related and neutral stimuli, leading to uniformly elevated drift rates.

Third, the absence of a congruency interaction may be developmentally mediated. Our sample consisted of adolescents and young adults, a group undergoing active maturation of prefrontal–limbic circuits (Corrigan et al. 2021; Rosenzweig et al. 2022). During this period, myelination of long‐range fronto‐limbic connections and fine‐tuning of inhibitory control mechanisms are still underway (Corrigan et al. 2021). This developmental window may render individuals more vulnerable to generalised attentional dysregulation and less likely to exhibit clearly dissociable attentional biases for discrete stimulus categories. Future studies should directly compare youth and older adults with insomnia to clarify the role of developmental neurobiology in modulating attentional dynamics.

### 4.4 Limitations

Several limitations warrant consideration. First, the cross‐sectional nature of the study precludes causal inferences regarding the directionality of attentional biases and symptom severity. Longitudinal designs could help disentangle whether sleep‐related attentional biases are antecedents or consequences of insomnia persistence.

Second, our dot‐probe task, while widely used, employed a relatively low trial count to maintain feasibility within a broader clinical protocol (Wiecki et al. 2013). Although HDDM is well‐suited to tasks with fewer trials, the precision of parameter estimates would benefit from higher trial densities in future work.

Third, no healthy control group was included. Although our study lacked a matched healthy control group, existing literature provides valuable behavioural benchmarks that can help contextualise our findings. For instance, meta‐analytic data indicate that individuals with insomnia or poor sleep exhibit a moderate attentional bias towards sleep‐related stimuli compared to healthy controls, corresponding to a Cohen's *d* = 0.44 (95% CI: 0.19–0.69) (Akram et al. 2023). A state‐of‐the‐science review has also highlighted evidence of sleep‐related attentional bias in poor sleepers, although effect sizes vary across tasks and studies (Harris et al. 2015). Compared to these behavioural norms (Table S4), our drift‐diffusion modelling revealed a statistically significant drift‐rate increase for congruent trials (*q* = 0.036), indicating accelerated evidence accumulation after the presentation of sleep cues, suggesting that our participants indeed show a computational analogue of the behavioural biases seen in prior research.

Given the absence of normative drift‐rate data in the literature, these cross‐study comparisons remain approximate. However, by aligning our findings with established behavioural benchmarks, we can cautiously infer that the attentional bias observed in our insomnia sample deviates meaningfully from expected normative patterns. We acknowledge this as a limitation, and while this was partially offset by stratification according to anxiety severity and the use of individual‐level parameter estimation, future studies should incorporate control comparisons to quantify the magnitude of attentional bias in insomnia relative to normative attentional processing.

Fourth, we focused exclusively on drift rate as the primary HDDM parameter of interest. While justified by theoretical considerations (Ratcliff and McKoon 2008; Ratcliff and Smith 2004), additional analyses examining threshold separation (parameter *a*), non‐decision time (*t*), or starting point bias (*z*) could offer complementary insights into the broader decision‐making profiles of insomnia patients.

An additional consideration relates to the clinical complexity of our sample. While the inclusion criteria ensured that all participants met diagnostic thresholds for insomnia disorder, a substantial proportion also met criteria for comorbid psychiatric conditions, particularly major depressive disorder and generalised anxiety disorder, as shown in Table S1. Although this reflects the clinical reality of insomnia presentations (Morin et al. 2015; Riemann et al. 2022), it may also introduce heterogeneity that could influence attentional processing and drift rate estimates. Future studies employing stratified designs or matched comorbidity profiles may help to isolate disorder‐specific cognitive mechanisms with greater precision.

Finally, our findings may not generalise beyond the narrow age range (15–24 years) sampled. Sleep regulation, attentional control and the prevalence of comorbid psychiatric conditions differ markedly across the lifespan. The developmental specificity of these findings should be investigated further using age‐diverse samples and potentially integrating puberty or hormonal status measures.

## 5 Conclusion

In summary, this study offers computational evidence for sleep‐related attentional bias in insomnia and highlights a dissociation between disorder‐specific and anxiety‐related attentional processes. Faster evidence accumulation when the probe appears in the location of sleep‐related words may reflect a maladaptive attentional pattern that sustains pre‐sleep arousal. In contrast, comorbid anxiety is associated with a generalised elevation in drift rate, consistent with diffuse hypervigilance. These findings advance our understanding of the cognitive‐affective mechanisms in insomnia and point towards the potential value of attention‐focused interventions, including cognitive bias modification and neuromodulation strategies, for mitigating hyperarousal and improving sleep outcomes.

## Author Contributions

**Isla Tsz Kwan Hui:** conceptualization, methodology, formal analysis, software, data curation, visualization, writing – original draft, writing – review and editing. **Tommy Ho‐Fung Chung:** methodology, investigation, data curation, writing – review and editing. **Sing‐Hang Cheung:** methodology, investigation, data curation, writing – review and editing. **Nazanin Biabani:** methodology, interpretation of results, writing – review and editing. **Panagis Drakatos:** interpretation of results, clinical expertise/validation, writing – review and editing. **Romola S. Bucks:** interpretation of results, writing – review and editing. **Sharon L. Naismith:** interpretation of results, writing – review and editing. **David O'Regan:** interpretation of results, writing – review and editing. **Veena Kumari:** conceptualization, supervision, writing – review and editing. **Dieter Riemann:** conceptualization, supervision, writing – review and editing. **Toby Wise:** methodology, software/computational modelling advice, formal analysis support, supervision, writing – review and editing. **Shirley Xin Li:** conceptualization, methodology, resources, supervision, project administration, funding acquisition, writing – review and editing. **Ivana Rosenzweig:** conceptualization, supervision, interpretation of results, writing – review and editing. All authors approved the final version and agree to be accountable for all aspects of the work.

## Funding

This work was supported by the General Research Fund of Research Grants Council, Hong Kong (17613321), the Wellcome Trust (225945/Z/22/Z), the NIHR Maudsley Biomedical Research Centre and the South London and Maudsley NHS Foundation Trust.

## Disclosure

The authors have nothing to report.

## Conflicts of Interest

The authors declare no conflicts of interest.

## Supporting information


**Table S1:** Demographic and clinical characteristics.
**Table S2:** Word stimuli used in the sleep‐related dot‐probe task, with strokes and frequency data in the Cantonese Chinese Language Corpus.
**Table S3:** Cantonese Chinese word stimuli used in the sleep‐related dot‐probe task, with strokes and frequency data in the Cantonese Chinese Language Corpus.
**Table S4:** Normative benchmarks for attentional bias measures in healthy controls versus the present insomnia sample.

## Data Availability

The data that support the findings of this study are available on request from the corresponding author. The data are not publicly available due to privacy or ethical restrictions.
