# Supplementary material for: Sleep‐Related Attentional Bias in Insomnia: A Drift Diffusion Model Approach
Source: J Sleep Res. 2026 Feb 24;35(4):e70315. doi: 10.1111/jsr.70315 (PMC13357999; doi:10.1111/jsr.70315)
Supplement: Supplementary file 1 — Table S1: Demographic and clinical characteristics. Table S2: Word stimuli used in the sleep‐related dot‐probe task, with strokes and frequency data in the Cantonese Chinese Language Corpus. Table S3: Cantonese Chinese word stimuli used in the sleep‐related dot‐probe task, with strokes and frequency data in the Cantonese Chinese Language Corpus. Table S4: Normative benchmarks for attentional bias measures in healthy controls versus the present insomnia sample. [file JSR-35-e70315-s001.docx]

SUPPLEMENT

| Table S1. Demographic and Clinical Characteristics.   \|  \|  \| **Low Anxiety** \| **High Anxiety** \| **M** \| **Total** \| ***p*** \| \| --- \| --- \| --- \| --- \| --- \| --- \| --- \| \| **N** \|  \| 64 (31.84%) \| 137 (68.16%) \|  \| 201 \| .675 \| \| **Age** \|  \| 20.14 (0.23) \| 20.01 (0.16) \| 6 \| 20.05 (0.13) \| .842 \| \| **Sex** \| Female \| 41 (64.06%) \| 74 (54.01%) \| 1 \| 115 \| .188 \| \|  \| Male \| 22 (34.38%) \| 63 (45.99%) \|  \| 85 \|  \| \| **Education** \| Secondary School \| 1 (1.56%) \| 5 (3.65%) \| 3 \| 6 \| .716 \| \|  \| University and above \| 62 (96.88%) \| 130 (94.89%) \|  \| 192 \|  \| \| **Medication** \| Nil \| 55 (85.94%) \| 109 (79.56%) \| 2 \| 164 \| .067 \| \|  \| Monotherapy \| 8 (12.5%) \| 16 (11.68%) \|  \| 24 \|  \| \|  \| Polytherapy \| 0 (0%) \| 11 (8.03%) \|  \| 11 \|  \| \| **HADS-A** \|  \| 5.20 (0.22) \| 11.34 (0.22) \| 0 \| 9.38 (0.26) \| <.001 \| \| **HADS-D** \|  \| 6.83 (0.44) \| 9.19 (0.23) \| 0 \| 8.438 (0.22) \| <.001 \| \| **ISI** \|  \| 14.78 (0.62) \| 16.18 (0.37) \| 0 \| 15.73 (0.32) \| .060 \| \| **MINI^a^** \| Nil \| 35 (54.69%) \| 33 (24.09%) \| 5 \| 68 \|  \| \|  \| Major depressive disorder \| 18 (28.13%) \| 58 (42.34%) \|  \| 76 \|  \| \|  \| Generalized anxiety disorder \| 8 (12.5%) \| 34 (24.82%) \|  \| 42 \|  \| \|  \| Dysthymia \| 4 (6.25%) \| 16 (11.68%) \|  \| 20 \|  \| \|  \| Social anxiety disorder \| 4 (6.25%) \| 7 (5.11%) \|  \| 11 \|  \| \|  \| Obsessive compulsive disorder \| 3 (4.69%) \| 5 (3.65%) \|  \| 8 \|  \| \|  \| Panic disorder \| 1 (1.56%) \| 4 (2.92%) \|  \| 5 \|  \| \|  \| Agoraphobia \| 2 (3.13%) \| 1 (0.73%) \|  \| 3 \|  \| \|  \| Post-traumatic stress disorder \| 0 (0%) \| 2 (1.46%) \|  \| 2 \|  \|   Data are presented as mean (standard error of the mean; SEM) for continuous variables and as count (percentage) for categorical variables. Group comparisons between participants with low versus high anxiety were conducted using Mann–Whitney U tests for continuous variables and Pearson’s χ² tests for categorical variables. M column– missing numbers.  ^a^ Participants could meet criteria for more than one current MINI diagnosis.  *Note*. Psychotropic medications (e.g., SSRIs) and other long-term non-psychotropic medications were permitted; medication status in Table S1 reflects any regular medication use recorded at baseline.  Table S2. Word stimuli used in the sleep-related dot-probe task, with strokes and frequency data in the Cantonese Chinese Language Corpus. | | | | | |
| --- | --- | --- | --- | --- | --- | --- | --- | --- | --- | --- | --- | --- | --- | --- | --- | --- | --- | --- | --- | --- | --- | --- | --- | --- | --- | --- | --- | --- | --- | --- | --- | --- | --- | --- | --- | --- | --- | --- | --- | --- | --- | --- | --- | --- | --- | --- | --- | --- | --- | --- | --- | --- | --- | --- | --- | --- | --- | --- | --- | --- | --- | --- | --- | --- | --- | --- | --- | --- | --- | --- | --- | --- | --- | --- | --- | --- | --- | --- | --- | --- | --- | --- | --- | --- | --- | --- | --- | --- | --- | --- | --- | --- | --- | --- | --- | --- | --- | --- | --- | --- | --- | --- | --- | --- | --- | --- | --- | --- | --- | --- | --- | --- | --- | --- | --- | --- | --- | --- | --- | --- | --- | --- | --- | --- | --- | --- | --- | --- | --- | --- | --- | --- | --- | --- | --- | --- | --- | --- | --- | --- | --- | --- | --- | --- | --- | --- | --- | --- | --- | --- | --- | --- | --- | --- | --- | --- | --- | --- | --- |
| Sleep words | Strokes | Frequency | Neutral words | Strokes | Frequency |
| tired | 11 | 459 | electricity | 13 | 659 |
| exhausted | 26 | 70 | utensil | 24 | <50 |
| fatigue | 23 | 77 | paddock | 20 | 108 |
| dream | 24 | 123 | plant | 26 | 347 |
| bed | 7 | 391 | red | 9 | 1825 |
| sheets | 19 | 54 | shuffle | 21 | <50 |
| wakeful | 22 | <50 | models | 23 | 435 |
| silence | 27 | 116 | texture | 28 | <50 |
| pillow | 24 | 124 | scents | 20 | 167 |
| naps | 16 | <50 | orange | 19 | 51/0 |
| dark | 25 | 490 | money | 26 | 56 |
| alert | 35 | <50 | vocal | 32 | 74 |
| snoring | 31 | <50 | machine | 32 | 1057 |
| overactive | 23 | <50 | absurd | 27 | 90 |
| arousal | 28 | 68 | impatient | 29 | 66 |
| sleepy | 17 | <50 | linen | 16 | <50 |
| night | 18 | 336 | quantifies | 16 | <50 |
| restless | 18 | <50 | youthful | 17 | 303 |
| tossing | 35 | 63 | authorize | 32 | 53 |
| lethargy | 21 | <50 | physics | 19 | 798 |
|  |  |  | leisure | 25 | <50 |
|  |  |  | poetry | 26 | 254 |
|  |  |  | welcome | 28 | 823 |
|  |  |  | prizes | 24 | <50 |
|  |  |  | gallant | 29 | 89 |
|  |  |  | joyful | 28 | 94 |
|  |  |  | outstanding | 29 | <50 |
|  |  |  | country | 21 | 10139 |
| Mean ± SD | 22.5 ± 7.104 | 118.55 ± 162.509 | Mean ± SD | 22.45 ± 6.557 | 317.790 ± 475.220 |

Note: The Cantonese Chinese Language Corpus reports frequency data only for words with an occurrence greater than 50; words occurring fewer than 50 times were recorded as zero for analytical purposes. In the original study, all stimuli were presented in Cantonese Chinese. The English translations shown here are provided for reference.

| Table S3. Cantonese Chinese word stimuli used in the sleep-related dot-probe task, with strokes and frequency data in the Cantonese Chinese Language Corpus. | | | | | |
| --- | --- | --- | --- | --- | --- |
| Sleep words | Strokes | Frequency | Neutral words | Strokes | Frequency |
| 累 | 11 | 459 | 電 | 13 | 659 |
| 疲憊 | 26 | 70 | 餐具 | 24 | <50 |
| 勞累 | 23 | 77 | 牧場 | 20 | 108 |
| 做夢 | 24 | 123 | 種植 | 26 | 347 |
| 床 | 7 | 391 | 紅 | 9 | 1825 |
| 床單 | 19 | 54 | 洗牌 | 21 | <50 |
| 無眠 | 22 | <50 | 模型 | 23 | 435 |
| 靜寂 | 27 | 116 | 質感 | 28 | <50 |
| 枕頭 | 24 | 124 | 氣息 | 20 | 167 |
| 小睡 | 16 | <50 | 橙子 | 19 | 51/0 |
| 黑暗 | 25 | 490 | 錢財 | 26 | 56 |
| 警醒 | 35 | <50 | 聲樂 | 32 | 74 |
| 鼻鼾 | 31 | <50 | 機器 | 32 | 1057 |
| 過勞 | 23 | <50 | 荒謬 | 27 | 90 |
| 喚醒 | 28 | 68 | 急躁 | 29 | 66 |
| 困倦 | 17 | <50 | 麻布 | 16 | <50 |
| 夜晚 | 18 | 336 | 量化 | 16 | <50 |
| 無休 | 18 | <50 | 青春 | 17 | 303 |
| 輾轉 | 35 | 63 | 授權 | 32 | 53 |
| 昏睡 | 21 | <50 | 物理 | 19 | 798 |
|  |  |  | 閒暇 | 25 | <50 |
|  |  |  | 詩歌 | 26 | 254 |
|  |  |  | 歡迎 | 28 | 823 |
|  |  |  | 獎品 | 24 | <50 |
|  |  |  | 華麗 | 29 | 89 |
|  |  |  | 歡快 | 28 | 94 |
|  |  |  | 優勝 | 29 | <50 |
|  |  |  | 國家 | 21 | 10139 |
| Mean ± SD | 22.5 ± 7.104 | 118.55 ± 162.509 | Mean ± SD | 22.45 ± 6.557 | 317.790 ± 475.220 |

Note: The Cantonese Chinese Language Corpus reports frequency data only for words with an occurrence count greater than 50; words with a frequency below this threshold were assigned a value of zero for analytical purposes.

**Table S4. Normative benchmarks for attentional bias measures in healthy controls versus the present insomnia sample.**

Normative values for dot-probe reaction time (RT)-based attentional bias represent pooled effect sizes from prior studies and meta-analyses comparing individuals with insomnia or poor sleep to healthy control participants (Akram et al. 2023). Positive Cohen’s *d* values indicate greater vigilance towards disorder-relevant stimuli relative to neutral stimuli. Drift-rate values (v) reflect the difference between congruent (sleep-related) and incongruent (neutral) trials derived from hierarchical drift diffusion modelling (HDDM) in the current study; no published normative drift-rate data for healthy controls performing sleep-related attentional bias tasks were identified. Cross-study comparisons should be interpreted cautiously, as methodological differences (task parameters, language, modelling approach) may influence absolute values. Nevertheless, alignment between our drift-rate effects and the magnitude/direction of RT-based biases in prior work suggests that the present sample exhibits a computational signature consistent with known deviations from normative attentional processing.

| Measure | Healthy Controls (from literature) | Current Insomnia Sample |
| --- | --- | --- |
| Dot‑probe RT-based attentional bias (Cohen’s d) | Moderate bias: d ≈ 0.44 (95% CI: 0.19–0.69) (Akram et al. 2023) | Small behavioural trend (paired RT p = .05265; n = 200) |
| Drift-rate difference (v_congruent vs. v_incongruent) | — (no published normative data) | Significant positive difference (q = .036) |
